# Spinal cord swelling and intradural compression predict neurological recovery after acute cervical traumatic spinal cord injury

**DOI:** 10.1371/journal.pone.0325827

**Published:** 2025-08-07

**Authors:** Harshit Arora, Hassan Darabi, Nathaniel Toop, Amy Minnema, Zahraa Al-Sharshahi, Grace M. Martin, Kelsey Karnik, Jan M. Schwab, Francis Farhadi

**Affiliations:** 1 Department of Neurosurgery, College of Medicine, University of Kentucky, Lexington, Kentucky, United States of America; 2 Department of Neurological Surgery, The Ohio State University Wexner Medical Center, Columbus, Ohio, United States of America; 3 Department of Biostatistics, University of Kentucky, Lexington, Kentucky, United States of America; 4 Department of Neurology and Belford Center for Spinal Cord Injury, The Ohio State University Wexner Medical Center, Columbus, Ohio, United States of America; Duke University Medical Center: Duke University Hospital, UNITED STATES OF AMERICA

## Abstract

Intradural spinal cord compression impairs perfusion pressure and is putatively rate-limiting for recovery after traumatic spinal cord injury (tSCI). After cervical tSCI, even minimally improved tissue preservation may help promote neurological recovery. To assess the nature and extent of spinal cord swelling and compression post-acute cervical tSCI, we evaluated several baseline MRI parameters including BASIC score, intramedullary lesion (IML) length, maximal canal compromise (MCC), maximal spinal cord compression (MSCC), extent of cord compression (ECC), maximal swollen anteroposterior diameter adjacent to injury site (Dmax), and maximal cord swelling (MCS) in 169 consecutive patients across 2 centers. In patients with either primarily intradural or combined (MSCC ≤5% or >5%, respectively) cord compression, we examined the predictive value of clinical and imaging admission parameters on American Spinal Injury Association Impairment Scale (AIS) severity and conversion up to 1-year follow-up. 37 (21.9%) patients presented with primarily intradural while 132 (78.1%) had combined cord compression. MSCC, MCS, and Dmax values differed significantly between the two groups (p < 0.0001, < 0.01 and < 0.001, respectively). MSCC was associated with age, MCC and MCS at baseline, while MCS was associated with age, MSCC and Dmax, on multivariable analysis. Logistic regression analysis of areas under receiver operating characteristic curve (AUROC) confirmed ECC (AUC 0.678) and MCS (AUC 0.922) as good and excellent predictors, respectively of AIS-conversion at 1-year for intradural compression participants. Additionally, MCS was significantly more accurate in predicting AIS-conversion in intradural group and the probability of AIS-conversion significantly decreased with each 1% increase in MCS (p = 0.003; OR 0.949), for both compression subtypes. In conclusion, baseline measures of cord swelling predict AIS-conversion likelihood up to 1-year. The deleterious effects of intradural cord compression, either isolated or presenting with extradural compression, may benefit from supplemental decompression strategies in addition to current standard-of-care.

## Introduction

The chronic functional impairment and disability resulting from tSCI place a considerable burden on individuals and their caregivers [1]. Along with varying degrees of motor, sensory, and autonomic dysfunction, multisystem early and delayed complications further complicate neurologic outcomes and increase mortality [2].

**Fig 1:**
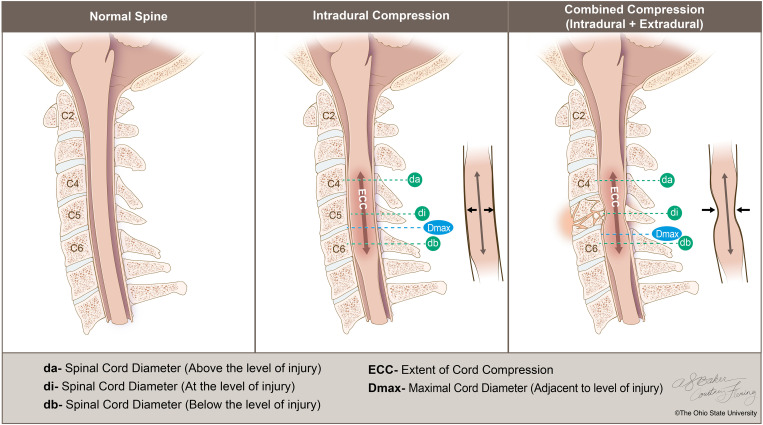
Artwork demonstrating the principal tSCI compression subtypes. The intradural subtype (shown in center) is characterized by primary spinal cord compression within the dura mater, underscoring that the contact on the spinal cord originates only from components contained in the intradural space. This internal pressure gives rise to a distinct localized egg-shaped appearance at the injury site, where the spinal cord diameter at the level of injury (di) is increased. With combined compression, there is both intradural compression and a discernible extrinsic pressure source (shown on right). This external compression suggests the involvement of factors lying outside the dura mater, such as bony fracture fragments, that significantly contribute to the spinal cord compression. The resultant combined compression typically results in an hourglass appearance where the diameter of the spinal cord at the injury level (di) is reduced as compared to the diameters above (da) and below (db) the injury. The extent of cord compression (ECC) measures the longitudinal span of spinal cord where both anterior and posterior cerebrospinal fluid signal are not evident. Dmax refers to the maximum anteroposterior spinal cord diameter adjacent to the site of spinal cord injury which, along with the da measurement, is used to calculate maximal cord swelling (MCS). Of note, the dashed lines refer to the levels at which the measurements are performed.

tSCI leads to varying degrees of local intraparenchymal spinal cord inflammation, progressive hemorrhagic necrosis, and swelling that potentially result in elevated intraspinal pressure (ISP). These processes in turn can compromise spinal cord perfusion pressure (SCPP), particularly when the cord swelling extends against the non-expansile dura mater, further reducing SCPP and potentially contributing to secondary injury [3]. This potential intradural compression is distinct from the extradural, or more precisely the combined spinal cord compression, that arises from fractures and/or dislocations of the spinal bony elements and the disco-ligamentous complex (see Fig 1).

Individuals with isolated intradural compression after tSCI offer a unique opportunity to analyze the evolution of spinal cord swelling, as the spinal cords in these circumstances are free from extradural constraints that separately limit cord expansion [4]. Careful distinction between these patterns of spinal cord compression is crucial, not only because it may allow for identification of baseline parameters with prognostic significance, but also because it may lead to the adoption of additional decompression strategies currently not included in the standard-of-care treatment regimen.

Despite decompression surgery being widely recognized as a mainstay intervention that can limit secondary injury, the exclusion of intradural or intraparenchymal decompression receives insufficient attention, in marked contrast to commonly-employed decompressive craniectomy protocols used in the management of traumatic brain injuries [5–7]. The use of measures aimed directly at the dura and spinal cord, such as expansion duroplasty and midline myelotomy have a longstanding history [3,8–12], and are currently being evaluated in a prospective trial [13]. Response to such additional measures may ultimately depend on the cord compression subtype, for instance with respect to the severity of intradural compression (either alone or when combined with extradural compression).

In this study, we combined independent tSCI cohorts from 2 academic institutions to identify the baseline demographic, clinical, and radiological characteristics of individuals who present with either primarily intradural versus combined (extradural and intradural) spinal cord compression, as evaluated on baseline standard-of-care MRI scans after tSCI. Using multivariable logistic regression analysis, we also (1) evaluate the association between these cord compression subtypes and other radiological measures of injury severity and (2) in a subset of subjects, we identify the combination of variables that are most predictive of neurological status and recovery.

## Materials and methods

### Study design

A retrospective cohort study was conducted across two academic level 1 trauma centers, the Ohio State University (OSU) and the University of Kentucky (UK). The study encompassed consecutive patients suspected of experiencing cervical spine trauma, spanning from January 2008 to May 2018 (OSU) and from May 2013 to May 2023 (UK). For OSU, as in a previous study [14], we used data from the institutional chapter of the Central Ohio Trauma System Registry (https://www.cotshealth.org/trauma) as well as from the Information Warehouse database while for UK we used the institutional trauma registry (https://ukhealthcare.uky.edu/doctors-providers/trauma). Long-term follow-up clinical evaluations were only available at OSU through participants’ electronic medical records and were primarily undertaken through scheduled visits with the Dodd SCI Rehabilitation Program, an awarded Center of the US-Spinal Cord Injury Model System. Ethical approval was obtained from the respective institutional review boards (protocol numbers 2015H0110 and 88464, respectively) and all guidelines were followed throughout the course of the study. Informed consent was waived given the retrospective nature of the study. Authors accessed electronic medical records for data collection and all individual participants’ data were de-identified prior to the start of data analysis. For the purposes of this research study, the data was accessed between August 15, 2020 and February 24, 2021 at OSU and between July 3, 2023 to March 11, 2024 at UK.

### Study participants

The study adhered to predefined inclusion and exclusion criteria. Consecutive patients presenting with acute cervical tSCI within the study period were considered for inclusion. The inclusion criteria were: 1) ≥18 years of age; 2) sustained either American Spinal Injury Association impairment scale (AIS) complete A or incomplete (B, C, D) SCIs; 3) presented with various clinical patterns of tSCI including central cord syndrome (CCS); 4) were either admitted directly or initially stabilized at other facilities and subsequently transferred for further acute management; 5) underwent AIS assessments and imaging within 72 h after injury. Exclusion criteria included: 1) concomitant traumatic brain or brachial plexus injuries making AIS assessments not reliable; 2) lack of clear evidence of spinal cord trauma with lesions related to nontraumatic causes (infection, tumor, vascular and metabolic); 3) AIS E tSCI; 4) prior surgical intervention on the cervical spine at an outside facility; 5) penetrating SCI (e.g., gunshot injuries); 6) institutional post-injury MRI absent or not performed within 72 hours.

### Data extraction

Various baseline parameters including the age on admission, sex, body mass index, and mechanism of injury were recorded. Injury mechanisms were classified into five categories, including motor vehicle collision (MVC), fall from height (high-energy), sports injury, mechanical fall (low-energy), and other high-energy trauma (OHT) that could not be classified otherwise into other groups. Injury and imaging times, injury severity score (ISS), Charlson Comorbidity Index (CCI), AIS on admission and on follow-up (when available), spinal fracture, presence of CCS, treatment details, length of stay (LOS), and in-hospital adverse events including death. Injuries were categorized according to the AIS scoring system with classifications ranging from AIS A – D indicating complete injury (A), incomplete injury with sensory sparing (B) and incomplete injury with motor sparing in less than and more than half the muscle groups below the level of injury (C and D, respectively) [15]. The neurological level of injury was defined as the most caudal spinal level at which all sensory and motor functions were normal.

### Radiological variables

Sagittal and axial T1 and T2-weighted cervical MRI views were used to measure and derive the various baseline parameters. These parameters included intramedullary lesion (IML) length, spinal canal diameters (Di, Da and Db), maximal canal compromise (MCC), maximum spinal cord compression (MSCC), spinal cord anteroposterior diameters (di, da, db, and Dmax), extent of cord compression (ECC), and maximal cord swelling (MCS) (see Fig 1 and Table 2) [3,16–19]. For each institutional series, two independent assessors that were blinded to the neurological outcomes performed the various measurements and the average values were used for the final analysis. Using axial T2-weighted views at the site of injury, the Brain and Spine Injury Centre (BASIC) [20] scores were also determined by two independent observers with any disagreements resolved by the senior author (who was also blinded to the neurological outcomes).

**Table 2:** Study imaging parameter descriptions and measurements (median).

		Intradural (n = 37)	Combined (n = 132)	Total	p-value
MCC (%)	*[(Da + Db)/2 – Di]/ (Da + Db)/2 x 100*	32.3 [17.4-42.0]	33.5 [17.7-53.3]	33.2 [17.6-51.2]	0.083
Di	*MS diameter of spinal canal at injury*	8.4 [7.3-10.3]	6.7 [5.5-8.6]	7.4 [5.8-8.9]	**< 0.0001**
Da	*MS diameter of the spinal canal above*	12.4 [10.6-13.5]	11.1 [9.6-12.5]	11.3 [9.8-12.8]	**0.002**
Db	*MS diameter of the spinal canal below*	13.2 [11.7-14.4]	11.4 [9.7-12.5]	11.8 [10.3-13.2]	**< 0.0001**
MSCC (%)	*[(da + db)/2 – di]/ (da + db)/2 x 100*	−6.1 [−19.1- −1.6]	22.9 [14.0-31.4]	17.4 [6.7-28.5]	**< 0.0001**
di	*AP cord diameter at injury*	7.3 [6.5-8.0]	5.0 [4.2-5.8]	5.5 [4.6-6.3]	**< 0.0001**
da	*AP cord diameter above*	6.9 [6.3-7.8]	6.7 [6.1-7.7]	6.8 [6.2-7.7]	0.373
db	*AP cord diameter below*	6.0 [5.4-6.8]	6.3 [5.8-6.9]	6.2 [5.7-6.9]	0.343
IML length (mm)	*Rostrocaudal length of intramedullary signal change*	26.8 [16.9-49.7]	23.4 [12.7-35.9]	24.1 [13.0-37.8]	0.161
BASIC score (n)					0.197
0	*No signal changes*	3	11	14	
1	*Signal change confined to central gray matter*	7	20	27	
2	*Signal changes extend beyond gray matter to involve white matter*	13	47	60	
3	*Signal changes involving entire transverse extent of spinal cord*	4	34	38	
4	*Grade 3 plus T2 hypointense foci of microhemorrhages*	10	19	29	
ECC (mm)	*Cord length without anteriorly and posteriorly surrounding CSF*	11.5 [7.7-21.0]	11.9 [5.2-23.3]	11.7 [5.6-22.2]	0.805
MCS (%)	*[(Dmax – da)/da] x 100%*	13.3 [4.8-23.0]	6.9 [−1.4-17.0]	7.7 [0.4-18.5]	**0.008**
Dmax	*AP maximal cord diameter at site adjacent to injury*	8.1 [7.3-9.4]	7.2 [6.5-8.1]	7.3 [6.5-8.4]	**0.0008**

AP, anteroposterior; BASIC, Brain and Spinal Injury Center; ECC, extent of cord compression; IML, intramedullary lesion; MCC, maximum canal compromise; MCS, maximal cord swelling; MS, midsagittal; MSCC, maximum spinal cord compression; IQR, interquartile range.

### Outcome variables

AIS grades of A and B (compared with AIS C and D) were defined as “severe neurological deficit.” Individuals with positive AIS conversion of at least one grade were grouped separately compared with those with no change or worsening in their AIS. Severe neurological deficit on admission as well as severe neurological deficit and AIS conversion on follow-up were analyzed as primary outcome variables.

### Statistical analysis

Continuous variables were reported as median with interquartile range (IQR) while frequency distributions were employed for categorical variables. Interclass correlation coefficients (ICC) were calculated for the radiological parameters for 66/169 patients as measures of interrater reliability [21]. Categorical variables were reported as the number of individuals within each cohort. The Shapiro-Wilk test was performed for all continuous variables to assess for normality of distribution. Comparisons between cohorts were conducted using the Mann-Whitney U test for continuous variables and Fisher’s Exact test for categorical variables, as the data did not follow a normal distribution. Spearman correlation was performed to assess the relationship between MSC, MSCC, ECC, Dmax and MCS. Univariate regression analysis was used to assess for the association between MSCC or MCS and the various baseline demographic and radiological measurements. A stepwise multivariable regression analysis was performed (including variables with p < 0.15 on univariate) to identify the relationship between the various baseline parameters and either MSCC or MCS. p < 0.05 was considered statistically significant. Further, to assess for multicollinearity, variance inflation factors (VIFs) were calculated for all included variables in the multivariable regression. Finally, Area under the Receiver Operating Characteristic (ROC) curve (AUC) analysis was employed to assess the association between the various radiological parameters and neurological severity rates, as well as AIS conversion rates, at 6-month and 1-year follow-up. All data were analyzed using R Studio Version 4.2.0 [22].

## Results

### Population characteristics

**Fig 2:**
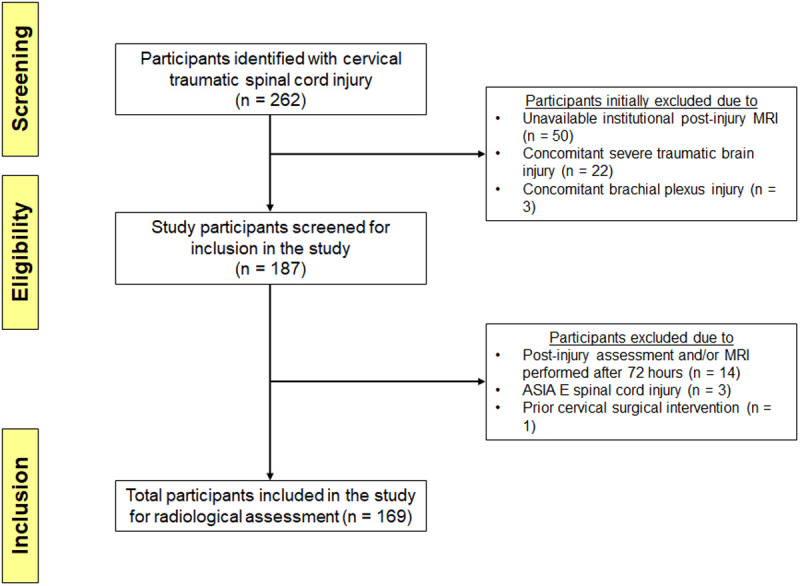
STROBE Flow Diagram Describing Study Cohort Selection.

A total of 262 participants were identified across the centers. Following screening and application of eligibility criteria, 169 individuals (103 from OSU, 66 from UK) and were included in the final analysis (see Fig 2). Overall, 37 (21.9%) were classified on admission MRI as exhibiting primarily intradural spinal cord compression and 132 (78.1%) with combined compression (Table 1). The Shapiro-Wilk test confirmed a non-normal distribution for the demographic variables and radiological parameters of the study participants. Baseline demographic factors including female/male sex ratio (7/30 vs 33/99, p = 0.517) and BMI (26.0 [IQR = 23.6–31.6] vs 28.0 [IQR = 24.1–31.9], p = 0.357) were not significantly different between groups. Injury mechanism was significantly different between groups (p = 0.013) with MVCs representing the most common form (n = 59), followed by mechanical falls (n = 50) and falls from height (n = 31). Admission injury severity scores (p = 0.173), AIS grades (p = 0.665), neurological level of injury (p = 0.444) and, the mean time-of-injury to MRI interval (9.1 [IQR = 6.1–19.1] vs 11.0 [IQR = 7.1–20.9] hrs, p = 0.230) were not significantly different between groups. Surgical decompression was performed in 147/169 (87.0%) patients and was achieved within 24 hours in 51/169 (30.2%) of cases, no significant differences were noted in surgery rates between groups.

**Table 1:** Baseline demographic and clinical characteristics of individuals admitted with cervical tSCIs (n = 169).

	Intradural (n = 37)	Combined (n = 132)	Total	p-value
Demographics				
Sex (female/male)	7/ 30	33/ 99	40/ 129	0.517
Age (median [IQR], yrs)	47.3 [28.0- 63.4]	55.4 [66.6-42.6]	54.0 [41.2-65.7]	**0.022**
BMI (median [IQR], kg/m^2^)	26.0 [23.6-31.6]	28.0 [24.1-31.9]	28.0 [23.7-31.7]	0.357
Comorbidities (CCI ≥ 1)	10	26	36	0.950
Injury mechanism				**0.013**
MVC	15	44	59	
Fall from height	6	25	31	
Mechanical fall	4	46	50	
Sports Injury	3	6	9	
OHT	8	11	19	
ISS				0.173
≤25	24	100	124	
>25	13	32	45	
AIS grade				0.665
A	11	32	43	
B	11	31	42	
C	3	15	18	
D	12	54	66	
Neurologic injury level				0.444
C4 and above	17	69	86	
C5 and below	18	53	83	
Time of Injury to MRI (hrs)	9.1 [6.1-19.1]	11.0 [7.1–20.9]	10.6 [6.9-20.2]	0.230
Surgery				0.776
No surgery	5	17	22	
≤24 hours	15	36	51	
>24 hours	13	79	92	
Delayed surgery (subsequent admission)	3	1	4	

tSCI, traumatic spinal cord injury; SD, standard deviation; MVC, motor vehicle collision; OHT, other high-energy trauma; ISS, injury severity score; AIS, American Spinal Injury Association impairment scale.

The flow diagram outlines the enrollment of study participants based on the screening process and the defined eligibility criteria.

### MRI parameters dichotomized by cord compression subtype

Table 2 displays statistical analyses comparing MCC, MSCC, IML length, BASIC score, ECC, and MCS between intradural and combined cord compression subtypes. The ICC revealed highest interrater reliability of measurements for ECC followed by average canal-based measurements (Di, Da, Db), average cord-based measurements (di, da, db), Dmax, and IML at 0.651, 0.645, 0.590, 0.569, and 0.565, respectively. These values are representative of moderate range interrater reliability [19]. Individuals with intradural cord compression had similar MCC as compared to those with combined cord compression injuries (32.3 [IQR = 17.4–42.0] vs 33.9 [IQR = 17.7–53.3], respectively; p = 0.083). However, lower Di, Da and Db variables were observed within the combined cord compression group (p = < 0.0001, 0.002 and < 0.0001, respectively). Likewise, the combined group had a significantly higher extent of MSCC (−6.1 [IQR = −19.1- −1.6] vs 22.9 [IQR = 14.0–31.4], p < 0.0001). The negative value indicates a spinal cord diameter that is increased from baseline at the level of injury. Rostrocaudal IML length was similar between cord compression groups (26.8 [IQR = 16.9–49.7] mm vs 23.7 [IQR = 12.7–35.9] mm, p = 0.161). BASIC score distributions, as assessed on axial T2-weighted views, were also not different between groups (p = 0.197). The most prevalent BASIC score was 2 in both groups, observed in 13/37 (35.1%) and 47/132 (35.6%) patients, respectively.

In contrast, significant differences were noted between groups in MCS (13.3 [IQR = 4.8–23.0] vs 6.9 [IQR = −1.4–17.0], p = 0.008) and associated Dmax values (8.1 [IQR = 7.3–9.4] vs 7.2 [IQR = 6.5–8.1], p = 0.0008), even though the ECC was not significantly different (11.5 [IQR = 7.7–21.0] mm vs 11.9 [[IQR = 5.2–23.3] mm, p = 0.805).

### Inter-relationships between baseline variables

**Fig 3:**
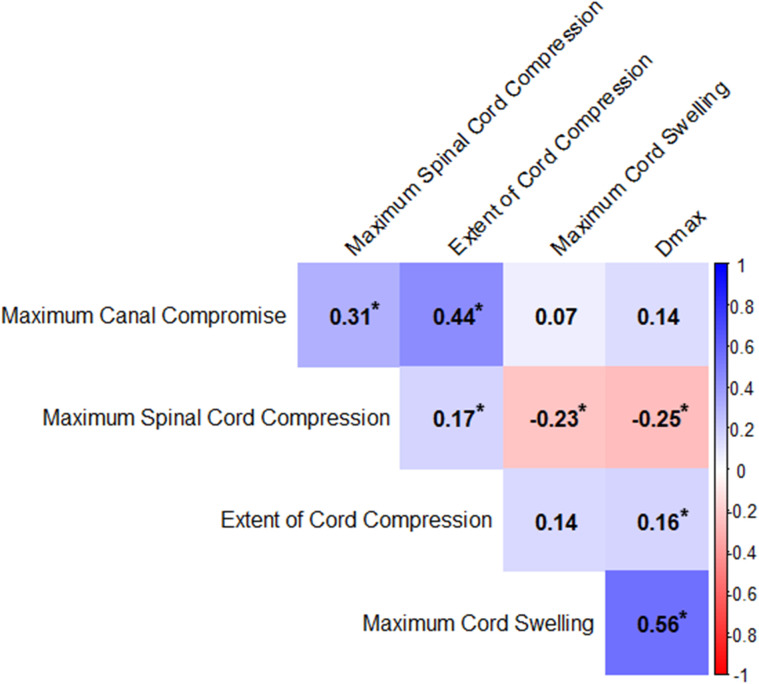
Spearman correlations between the various baseline radiological parameters. Correlation heat-plot matrix depicting the Spearman correlation coefficients (r) for the various radiological variables assessed in the study. Blue intensity corresponds to the degree of positive correlation (r > 0) while red intensity corresponds to the degree of negative correlation (r < 0). No correlation is represented by white (r = 0). Significant associations between two variables (p < 0.05) are represented with asterisks (*) in the respective cells.

Spearman correlations were performed to explore potential relationships between the various baseline radiological parameters (Fig 3). The analysis shows a strong correlation between MCS and Dmax (r = 0.56, p ≤ 0.0001). A weak positive correlation is noted between MSCC and ECC (r = 0.17, p = 0.032). Negative correlations are shown between MSCC and MCS (r = −0.23, p = 0.003) and MSCC and Dmax (r = −0.25, p = 0.001).

Regression analyses were conducted to analyze the association between various baseline variables and either MSCC or MCS (Table 3). Significant associations are shown on multivariable analysis between MSCC and age (p < 0.0001), MCC (p = 0.005), MCS (p = 0.0002), and ECC (p = 0.032). With respect to MCS, significant associations on multivariable analysis are shown for MSCC (p = 0.0001) and Dmax (p = 0.048). Further, the VIFs for all variables across both regression models were all < 2, indicating no evidence of multicollinearity.

**Table 3:** Significant inter-relationships between baseline variables and MSCC or MCS.

	MSCC				MCS			
	Univariate Analysis		Multivariable Analysis		Univariate Analysis		Multivariable Analysis	
	Coefficient (95% CI)	p-value	Coefficient (95%CI)	p-value	Coefficient (95%CI)	p-value	Coefficient (95%CI)	p-value
**Age**	0.41 (0.23- 0.58)	**<0.0001**	0.37 (0.2-0.53)	**<0.0001**	–	–	–	**–**
**IML**	−0.16 (−0.03-0.015)	0.075	−0.08 (−0.27-0.11)	0.395	0.36 (0.21-0.51)	**<0.0001**	0.02 (−0.17-0.21)	0.854
**MCC**	0.29 (0.138-0.45)	**0.0003**	0.22 (0.06-0.37)	**0.005**	–	–	–	–
**MSCC**	–	–	–	–	−0.32 (−0.45 - −0.20)	**<0.0001**	−0.21 (−0.33 - −0.08)	**0.0001**
**MCS**	−0.39 (−0.55 - −0.24)	**<0.0001**	−0.34 (−0.51 - −0.16)	**0.0002**	–	–	–	–
**ECC**	0.22 (0.00-0.44)	0.051	0.25 (0.02-0.48)	**0.032**	0.22 (0.02-0.42)	**0.034**	0.06 (−0.13-0.26)	0.520
**Dmax**	−4.59 (−6.8 - −2.37)	**<0.0001**	−1.34 (−3.95-1.26)	0.311	8.18 (6.47-9.89)	**<0.0001**	6.63 (4.63-8.64)	**0.048**

† MSCC = Maximal spinal cord compression; MCS = Maximal cord swelling; CI = Confidence Interval; IML = intramedullary length; MCC = maximum canal compromise; ECC = extent of cord compression; dmax = Maximal diameter of spinal cord above the site of injury.

To determine whether there is a relationship between cord swelling and time of injury to MRI, individuals were stratified into subgroups based on whether the MRI study was performed ≤ 12 hrs, between 12–24 hrs, or between 24–72 hrs from the time of injury. While no significant differences on ANOVA were observed in either Dmax or MCS, ECC was significantly different across the three defined time intervals (p = 0.025).

### Baseline MRI variables and outcome analysis

Follow-up AIS evaluations were available at 6 months and at 1-year post-injury intervals for a total of 103 individuals (see Table 4 and Supplementary Fig 1). No differences were noted between tSCI compression subtypes in mortality or follow-up rates (p = 0.678). All baseline MRI variables were analyzed for their ability to predict neurological severity (Table 5) and AIS conversion (Table 6) within both the intradural and combined compression tSCI groups using AUC analysis. Each variable’s predictive strength was analyzed and compared for both the intradural and combined compression tSCI groups at both follow-up time points. MCC and MSCC are poor predictors (AUC range 0.294 to 0.653) while ECC is a fair predictor (AUC range 0.659 to 0.824) of neurological severity for both compression groups. In contrast, MCS is a good predictor (AUC range 0.700 to 0.850) while IML length and BASIC are excellent predictors (AUC ≥ 0.889) at both 6 months and 1-year. There were no significant differences with respect to the neurological severity predictive abilities of these various measures at the two follow-up intervals.

**Table 4:** Follow-up for individuals with acute cervical tSCI (n = 103).

	Intradural	Combined	Total	p = 0.678
Minimum 1-year follow-up	20	52	72	
Death during initial admission	3	5	8	
Death at 1-year follow-up	5	10	15	
Loss to follow-up	1	7	8	

A chi-square test was performed to evaluate the statistical difference between the intradural and combined group outcomes at one-year follow-up.

**Table 5:** Area under the ROC curve (AUC) analysis to predict neurological severity at 6-months and 1-year following acute cervical tSCI.

	Neurological Severity at 6-months			Neurological Severity at 1-year		
	Intradural	Combined	p- value	Intradural	Combined	p- value
MCC	0.435	0.647	0.227	0.463	0.653	0.289
MSCC	0.294	0.558	0.122	0.300	0.601	0.083
IML length	0.906	0.889	0.886	0.913	0.896	0.884
BASIC	1.000	0.889	0.276	1.000	0.923	0.375
ECC	0.659	0.782	0.449	0.687	0.824	0.383
MCS	0.824	0.700	0.451	0.850	0.741	0.495

BASIC, Brain and Spinal Injury Center; ECC, extent of cord compression; IML, intramedullary lesion; MCC, maximum canal compromise; MSCC, maximum spinal cord compression; MCS, maximal cord swelling; ROC, receiver operating characteristic.

**Table 6:** Area under the ROC Curve (AUC) analysis to predict AIS conversion at 6-months and 1-year following acute cervical tSCI.

	AIS Conversion at 6-months			AIS Conversion at 1-year		
Group	Intradural	Combined	p- value	Intradural	Combined	p- value
MCC	0.487	0.641	0.298	0.411	0.623	0.195
MSCC	0.308	0.604	**0.036**	0.233	0.611	**0.011**
IML length	0.795	0.722	0.533	0.956	0.840	0.097
BASIC	0.774	0.788	0.905	0.950	0.856	0.177
ECC	0.641	0.672	0.824	0.678	0.713	0.808
MCS	0.889	0.692	**0.048**	0.922	0.700	**0.018**

BASIC, Brain and Spinal Injury Center; ECC, extent of cord compression; IML, intramedullary lesion; MCC, maximum canal compromise; MSCC, maximum spinal cord compression; MCS, maximal cord swelling; ROC, receiver operating characteristic.

Similar findings are noted for these MRI variables as predictors of AIS conversion with the intrinsic cord-signal and swelling-based values again performing more robustly as compared to MCC and MSCC. While MCC and MSCC again performed poorly overall (AUC range 0.233 to 0.641), the predictive ability of MSCC is significantly better for individuals with combined compression at both the 6-month (p = 0.036) and 1-year (p = 0.011) follow-up time points. Once again, while ECC is a fair predictor (AUC range 0.641 to 0.713), IML length and BASIC are excellent predictors particularly at 1-year follow-up (AUC range 0.840 to 0.956). In contrast to all the other robust predictors of AIS conversion, MCS performed significantly better at predicting AIS conversion for individuals with primarily intradural compression at both the 6-month (0.889 vs 0.692, p = 0.048) and 1-year follow-up time points (0.922 vs 0.700, p = 0.018).

Finally, we performed binary ordinal logistic regression analysis to evaluate the probability of AIS conversion based on ECC and MCS. For both compression subtypes, we observe that the chance of AIS conversion significantly decreases with every 1 mm increase in ECC (p = 0.004; OR 0.938) and with each 1% increase in MCS (p = 0.003; OR 0.949).

## Discussion

Secondary biochemical injury processes after tSCI begin immediately and progress over hours and days [23]. This phase is characterized by a wide range of pathophysiological mechanisms including progressive hemorrhagic necrosis, inflammation, reduced blood flow, toxic effects from excessive glutamate release, ionic imbalance, and oligodendrocyte death [24–26]. Spinal cord swelling, an integral component of secondary injury [27], leads to elevated ISP and reduced SCPP, potentially further compounding injury severity [28–30].

Given shared pathologic CNS injury mechanisms involving edema and elevated intercompartment pressure [12,31], standard-of-care management for both traumatic brain injury and tSCI include decompressive surgical techniques including decompressive hemicraniectomy, discectomy, corpectomy, and laminectomy. However, while cranial procedures typically include opening and enlargement of the dural compartment (expansive duroplasty), this component has not historically been included in spinal procedures. Incorporation of more aggressive options like myelotomy and expansive duroplasty into the management stream remains a matter of debate and requires further evaluation [10,32]. This concept is clearly worth exploring, given our decades-long use and proven benefit of decompressive hemicraniectomies in TBI care, which always involve dural opening (Fig 1) [12,13,33,34].

In this paper, we delineate two distinct compression patterns of tSCI to explore the related differences in canal and spinal cord-based measurements. In line with previous work [9], we distinguished tSCIs based on their corresponding MSCC ratios; a ≤ 5% cutoff was used to define intradural compression while all other injuries were assigned to the combined group (see Fig 1). To our knowledge, our study presents the first analysis of the relationship between these compression subtypes (dural-only versus combined dural and extrinsic/ discoligamentous) and neurological outcomes. This dichotomization also allows for a novel evaluation of distinct cord-based measures of swelling applying standard MRI techniques. Specifically, Dmax and MCS have been recognized as relevant measures of the degree of anteroposterior cord swelling [18]. Both measures are significantly greater and are more robustly predictive of neurologic recovery specifically in the intradural group. Of note, these two measures showed a significant inverse correlation with MSCC (Fig 3). This is in line with the observation that the intradural group has higher Dmax values than the combined group given the absence of extrinsic compression that allows the spinal cord to swell further in the anteroposterior dimension. As such, we conclude that the absence of extrinsic compression is relatively more permissive of the injured spinal cord’s tendency to swell post-tSCI and thus allows for detection of the relevance of the associated secondary injury processes in predicting neurologic recovery. In our cohort, combined compression apparently did not allow for a similar degree of cord swelling despite a similar profile of underlying tSCI mechanisms and severity on admission.

ECC is a measure of cord swelling in the longitudinal axis [9], and may therefore similarly track post-SCI intraparenchymal changes as compared to Dmax and MCS. However, while we failed to identify a significant correlation between ECC and MCS (p = 0.065), a significant negative correlation was identified between ECC and MSCC (p = 0.032) in our cohort. Further, ECC is not significantly different between the compression subtypes in our study. This observation actually aligns with prior studies that demonstrated that the extent of cord swelling, for which ECC serves as a surrogate, may correlate with injury severity following tSCI [35,36]. Since injury severity on admission (e.g., baseline ISS and AIS) is similar between our two study groups, it appears that this longitudinal-based measure does not as faithfully track cord swelling as compared to the injury site axial-based Dmax and MCS measures.

To our knowledge, our study also suggests for the first time that younger patients are more prone to develop primarily intradural compressive cervical tSCI (see Table 1). This may in part be attributable to the tendency of older patients to suffer from fractures and/or dislocations of the spine [37,38], potentially manifesting as central cord syndrome in cases with pre-existing cervical spondylosis with spinal canal narrowing and spinal cord compression (see Table 3). As the age demographic of tSCI is changing over time [39,40], we believe that this important compression subtype may remain underrecognized.

IML length and BASIC scoring have emerged as effective surrogate markers for assessing the severity of tSCI, based on sagittal and axial imaging characteristics, respectively [14,16,20]. In our cohort, these measures demonstrated significant utility for predicting neurologic severity and AIS conversion for both compression subtypes up to one-year follow-up. The one-year mark is pivotal for individuals with tSCI, as it generally marks the transition to a chronic recovery state following which significant recovery is less likely [41]. In line with the critical importance of mid-sagittal “tissue bridges” to enable neurological recovery, these findings underscore the relevance of early neuroprotection strategies though sufficient spinal cord decompression [14,42]. ‘Time (to decompression) is spine’ relates to an overarching paradigm mirroring the span of time over which the swollen spinal cord is strangulated by its local environment. Here, we propose and validate pragmatic, real-world MRI measures to assess different phenotypes of spinal cord swelling being predictive of neurologic recovery after tSCI. The represented MRI-markers characterizing post traumatic edema are also candidates to further improve stratification for interventional trials and prognostication models.

Consistent with the most recent guidelines [43,44], many trauma centers have adopted an early decompression surgical strategy in their management of patients with acute tSCI. While the benefits of this approach are increasingly recognized, it may lead to underappreciation of the extent of spinal cord swelling that is expected to reach a peak stage at 3–5 days after injury [9,45–47]. In our study, a significantly greater proportion of subjects with combined compression underwent surgery ≥24 hours post-injury, which may be attributed to both extrinsic (e.g., logistical or transportation-related limitations) and intrinsic factors (e.g., need for stabilization). To assess for a potential relationship between the progression of spinal cord swelling and time of injury, we categorized the study participants into 3 groups based on their injury-MRI time intervals and identified significant time-dependent differences in ECC measurements. Further controlled studies will be needed to confirm this finding and more accurately define the timescale of cord swelling following acute tSCI.

Taken together, our study supports the notion that intradural compression remains an underappreciated source of injury to the spinal cord that may independently affect outcomes. The reliability of our study findings is supported by the relatively large number of patients included over two independent trauma centers. Potential selection bias was minimized through the establishment of uniform criteria and standardized data collection procedures. Another potential limitation is the moderate inter-rater reliability observed for spinal cord radiological measurements. While these measurements support the overall trends and findings, the moderate agreement may introduce some degree of variability. This may in turn impact the precision or reliability of the results, underscoring the need for future studies with larger sample sizes to assess these radiological variables in acute cervical tSCI and their clinical associations. Among the 169 patients, 37 (21.9%) had primarily intradural compression at baseline, which is consistent with the work of Saadoun and colleagues (25.8%) [9] but higher than the estimate provided by Aarabi and colleagues [10] when evaluating the proportion of patients that underwent “inadequate decompression” with noted absence of cerebrospinal fluid on post-operative imaging (9.6%). An ongoing clinical trial evaluating the potential benefits of expansive duroplasty may shed further insight into the clinical relevance of intradural spinal cord compression [13].

## Conclusion

This study reveals the presence of a distinct group of individuals with tSCI who experience spinal cord compression primarily related to swelling within the intradural compartment without associated significant compromise of the spinal canal. This distinct cohort highlights the clinical relevance of baseline Dmax and MCS measures as additional predictors of the likelihood of neurological recovery after tSCI. Since spinal cord swelling may be variably noted in both tSCI cord compression modes, our findings provide a rationale for careful evaluation of these measures during the first few days after tSCI. More importantly, our findings suggest that individuals with tSCI may benefit from additional decompression of the intradural compartment by mitigating the secondary injury associated with intradural cord compression and ischemia. Further confirmation of these findings over time will provide a rationale for a tailored surgical strategy as defined by the patient-specific pathophysiological tSCI features.

## Supporting information

Supplementary Fig 1 Relationship of cord compression subtype with neurologic recovery over the first year after tSCI. Neurological recovery profiles after tSCI of individual subjects (represented as blue lines) are displayed at 6 and 12 months after injury in comparison to baseline AIS scores for both intradural (left) and combined (right) compression subtypes.
(TIF)
