# Supplementary figures and images for: Spinal cord swelling and intradural compression predict neurological recovery after acute cervical traumatic spinal cord injury
Source: PLoS One. 2025 Aug 7;20(8):e0325827. doi: 10.1371/journal.pone.0325827 (PMC12331075; doi:10.1371/journal.pone.0325827)

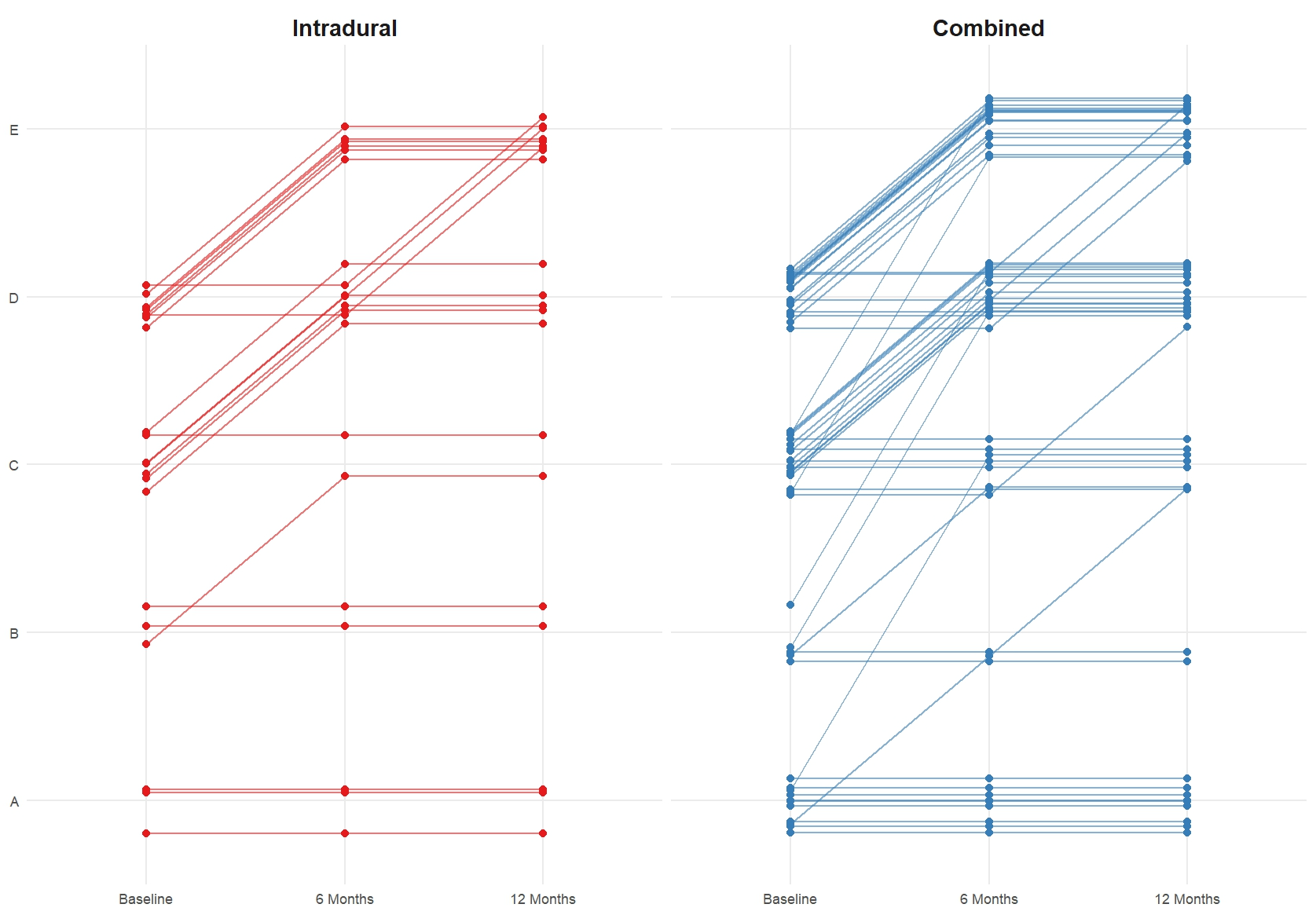

Supplement: Supplementary Fig 1 — Neurological recovery profiles after tSCI of individual subjects (represented as blue lines) are displayed at 6 and 12 months after injury in comparison to baseline AIS scores for both intradural (left) and combined (right) compression subtypes. (TIF) [file pone.0325827.s001.tif]
